# From ribosomopathies to therapeutic targets: ribosomal alterations in pediatric leukemogenesis and tumorigenesis

**DOI:** 10.3389/fonc.2026.1832877

**Published:** 2026-06-19

**Authors:** Michalina Horochowska, Ewa Jakubczyk, Marek Ussowicz

**Affiliations:** 1 Department of Pediatric Bone Marrow Transplantation, Oncology and Hematology, Wroclaw Medical University, Wrocław, Poland; 2 Department of Pediatric Bone Marrow Transplantation, Oncology and Hematology, University Clinical Hospital, Wrocław, Poland

**Keywords:** Diamond–Blackfan anemia, leukemia predisposition, medulloblastoma, neuroblastoma, nucleolar stress, p53, ribosomal proteins, ribosome biogenesis

## Abstract

Ribosomes are central to cellular growth and proteome maintenance, and cancer cells frequently depend on increased ribosome biogenesis and altered translation to sustain proliferation, stress tolerance, and metabolic rewiring. Paradoxically, inherited “ribosomopathies” caused by germline defects in ribosomal proteins or ribosome biogenesis factors present with tissue hypoplasia and bone marrow failure early in life, yet confer substantially increased lifetime risk of myelodysplastic syndrome (MDS), acute myeloid leukemia (AML), and selected solid tumors. In parallel, somatic alterations affecting ribosomal proteins and ribosome regulatory pathways recur across malignancies, including hematologic cancers such as T-cell acute lymphoblastic leukemia (T-ALL), and in pediatric solid tumors. Mechanistically, oncogenic ribosome disturbances can reprogram translation toward specific mRNA subsets, alter translational fidelity, trigger nucleolar/ribosomal stress signaling via the 5S ribonucleoprotein (5S RNP)–MDM2–p53 axis, and enable selection for compensatory or cooperating lesions (notably TP53 pathway alterations). Clinically, these insights support diagnostics and surveillance of inherited ribosome-related cancer predisposition syndromes, therapeutic targeting of ribosome biogenesis and translational control, including RNA polymerase I inhibition, and the eIF4 translation-initiation machinery. Here, we present current evidence linking constitutional and acquired ribosomal dysfunction to leukemogenesis and tumorigenesis, highlight disease- and context-specific mechanisms, and outline priorities for translational research and precision therapy.

## 1 Introduction

Ribosomes integrate growth signals, nutrient availability, and stress responses into protein synthesis. In eukaryotes, ribosome production is a resource-demanding, multi-compartment process requiring rRNA transcription (primarily by RNA polymerase I), rRNA processing and chemical modification, coordinated expression and nuclear import of approximately 80 ribosomal proteins (RPs), and stepwise assembly and cytoplasmic export of pre-40S and pre-60S particles through the nucleolus, nucleoplasm, and cytoplasm. Because this machinery sits at the intersection of cell growth and quality control, it is tightly coupled to tumor suppressor networks—most prominently the MDM2–p53 axis.

Cancer cells commonly exhibit nucleolar enlargement and increased ribosome biogenesis, reflecting dependence on elevated rRNA synthesis and translational output.[9] Contemporary evidence indicates that hyperactive ribosome biogenesis contributes not only to proliferation but also to cancer stem-like states, epithelial–mesenchymal transition (EMT), and metastasis, making ribosome production a tractable therapeutic vulnerability (1).

**Figure 1:**
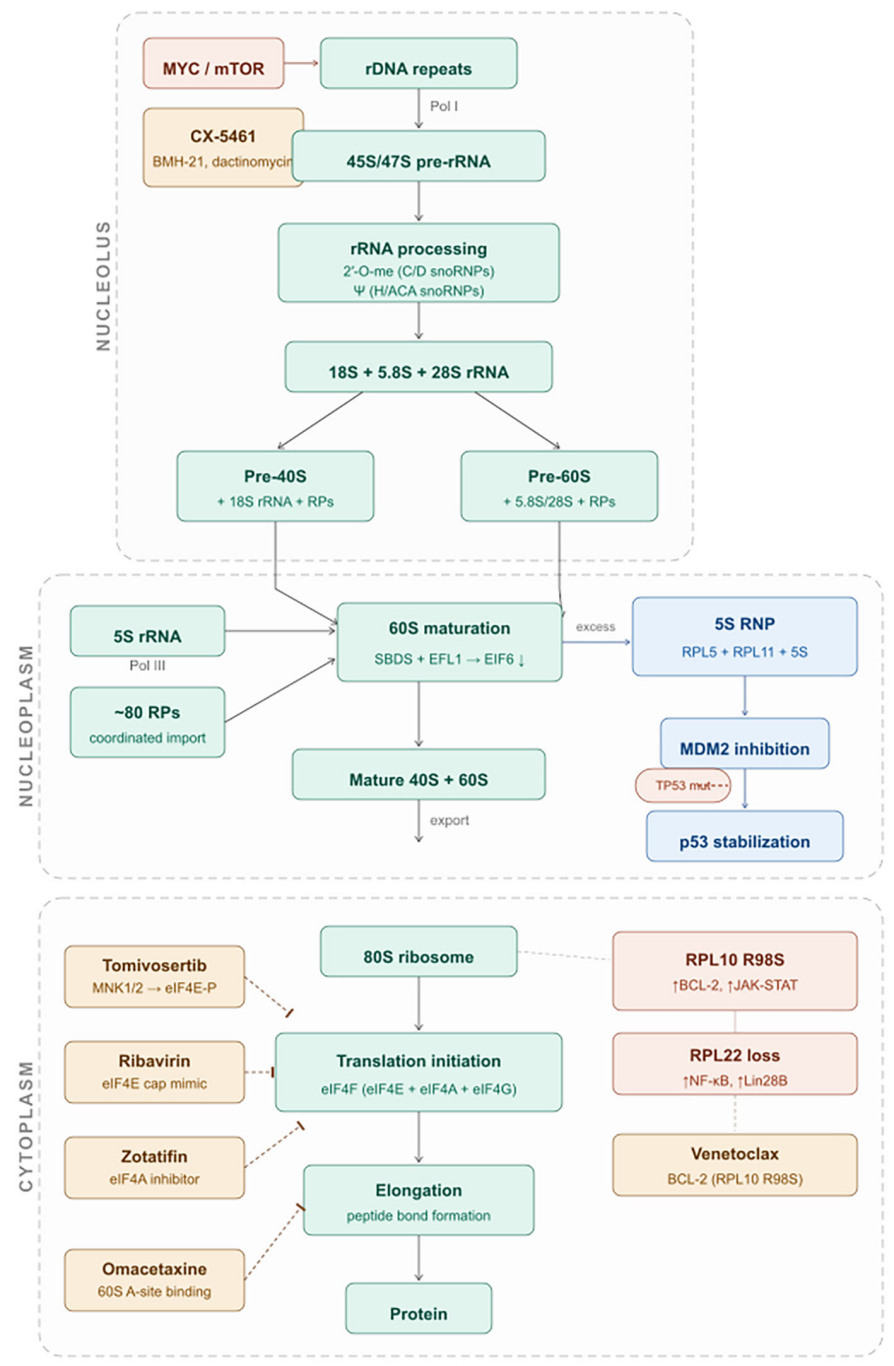
Ribosomopathy paradox: from ribosomal defect to leukemogenesis and tumorigenesis.

A defining conceptual challenge is the “ribosomopathy paradox”: germline defects in ribosomal proteins or assembly factors cause hypo-proliferative phenotypes (such as bone marrow failure), yet predispose to later malignant transformation. This paradox is increasingly explained by a two-phase model (**Figure 1**). In phase 1, a ribosomal lesion impairs biogenesis, and excess free 5S ribonucleoprotein inhibits MDM2, stabilizing p53 and driving lineage-specific apoptosis. In phase 2, sustained p53-driven pressure bifurcates clonal evolution, compensatory clones acquire ribosome-rescuing mutations that relieve p53 activation and carry low leukemic potential, whereas pre-malignant clones disable the checkpoint through *TP53*, del(17p), or *MDM2*/*MDM4* alterations, unleashing pro-oncogenic translational reprogramming that drives transformation.

Understanding this sequence is essential for risk stratification, surveillance, and therapeutic intervention in patients with inherited ribosomopathies.

Throughout this review, we use the following terms consistently. “Ribosomopathy” denotes a syndrome caused by a germline or, in the case of del(5q), an acquired constitutional loss-of-function mutation in a ribosomal protein gene or ribosome biogenesis factor, resulting in a primary hypo-proliferative phenotype. “Ribosome biogenesis stress” refers to any perturbation—constitutional or somatic, loss- or gain-of-function—that imbalances the stoichiometry of ribosome assembly intermediates. “Nucleolar stress” (a term used interchangeably with “ribosomal stress” in the literature) specifically describes the activation of the 5S RNP–MDM2–p53 surveillance axis that results from such imbalance. “Translation dysregulation” encompasses qualitative changes in translational output—including altered IRES usage, ribosome specialization, modified translational fidelity, and oncogene-driven, cap-dependent translation enhancement—that may occur independently of ribosome biogenesis. Primary ribosomopathies and MYC-driven ribosome biogenesis addiction represent opposite ends of this spectrum: the former is defined by ribosome insufficiency and constitutive nucleolar stress, and the latter by ribosome excess that paradoxically requires intact p53 pathway escape for a full oncogenic effect.

The present review focuses on three themes: (1) constitutional mutations and dysfunction (ribosomopathies and related cancer predisposition states); (2) somatic mutations in ribosomal proteins and ribosome-associated genes found recurrently across leukemias and solid tumors; and (3) functional dysregulation of ribosome biogenesis and translation as a targetable hallmark in leukemogenesis and tumorigenesis.

## 2 Literature search and scope

We performed a narrative review using PubMed, Embase, and open-access full texts. Search terms included combinations of “ribosomopathy”, “ribosomal protein mutation”, “ribosome biogenesis cancer”, “nucleolar stress”, “Diamond-Blackfan anemia cancer”, “Shwachman-Diamond syndrome leukemia”, “RPL10”, “RPL22”, “RPL5”, “RPS15”, “RPS14”, “RNA polymerase I inhibitor”, “CX-5461”, “eIF4 cancer”, and related terms. We prioritized: cohort and registry studies in inherited bone marrow failure syndromes with ribosomal involvement, studies of recurrent somatic ribosomal protein mutations in hematologic malignancies and solid tumors, pan-cancer analyses and functional genomics datasets for ribosomal protein alterations, and clinical and translational studies targeting ribosome biogenesis or translation, including early-phase trials. Key recent systematic reviews and meta-analyses were used as organizing milestones. No language or date restrictions were applied. The literature search for this review was last updated on 6 March 2026.

Publications were selected on the basis of relevance to ribosome biology, ribosomopathies, somatic ribosomal alterations, and therapeutic targeting in cancer, with priority given to studies focused on pediatric malignancies, landmark mechanistic papers, recent high-impact articles, and clinically informative translational studies. In areas where pediatric evidence remains limited, adult studies were included when they offered strong biological rationale or potential relevance for future pediatric investigation. This manuscript should be regarded as a narrative, not systematic, review, and the included literature was curated to summarize the field in a focused and interpretive manner rather than through a formal systematic-review methodology.

## 3 Ribosome biogenesis, translational control, and surveillance pathways in cancer

### 3.1 Ribosome biogenesis as a growth checkpoint

Ribosome biogenesis couples oncogenic signaling to cell-cycle progression through multiple converging pathways. Key oncogenes—most prominently *MYC* and the *PI3K*–*AKT*–*mTOR* axis—directly stimulate rRNA transcription by RNA polymerase I and upregulate the expression of ribosomal protein genes and translation-related factors (2). Tumors dependent on elevated Pol I activity show cancer-specific characteristics that may be exploited pharmacologically.

Conversely, tumor suppressors such as *RB*, *PTEN*, and *ARF* restrain these outputs through transcriptional and post-translational mechanisms. Increased ribosome biogenesis is now recognized as a feature in many cancers and a driver of invasive and metastatic traits rather than merely a passive correlate of proliferation (3).

The rate-limiting step of rRNA synthesis—transcription of the 45S/47S precursor rRNA from multiple tandem repeat units (rDNA) in the nucleolus—is controlled by the Pol I pre-initiation complex, which includes UBF1/2, SL1, and RRN3/TIF-IA (4). *MYC*-driven transcriptional programs activate Pol I, while energy and amino acid limitation signal through AMPK and mTOR to suppress it (5, 6). This relationship with metabolic stress can be exploited pharmacologically by Pol I inhibitors.

### 3.2 Nucleolar/ribosomal stress and the 5S RNP–MDM2–p53 axis

A consequence of impaired ribosome production is nucleolar stress, which stabilizes p53 through the 5S ribonucleoprotein (5S RNP) pathway. When ribosome biogenesis is disturbed, 5S RNP complexes—composed of 5S rRNA, *RPL5* (uL18), and *RPL11* (uL5)—accumulate outside assembling ribosomes and bind the RING domain of MDM2, competitively inhibiting its E3 ubiquitin ligase activity toward p53 (7, 8). Structural and biochemical studies have refined our understanding of how nascent 5S RNP is assembled co-transcriptionally and how specific RP mutations can uncouple this surveillance mechanism (7). The 5S RNP–MDM2–p53 pathway enforces growth restriction and lineage-specific apoptosis in response to ribosome assembly defects—explaining the hypoplastic phenotypes of ribosomopathies (9). In cancer evolution, however, cells that acquire *TP53* pathway alterations (*TP53* mutation, *MDM2* amplification, or *MDM4* overexpression) can escape this checkpoint. This co-occurrence pattern provides strong genetic evidence that ribosomal gene loss creates selective pressure for *TP53* pathway co-mutation, directly paralleling the clonal evolution dynamics observed in inherited ribosomopathies such as SDS and DBA (3, 10). Synthesizing these findings yields a unified mechanistic cascade (**Figure 1**) that connects ribosome biogenesis defects to leukemogenesis and tumorigenesis: 1. a ribosomal lesion—constitutional (RP haploinsufficiency, *SBDS* loss, or *DKC1* loss) or somatic (del(5q)/*RPS14* loss or acquired RP mutations)—impairs ribosome biogenesis; 2. excess free 5S RNP accumulates and inhibits *MDM2*, stabilizing p53; 3. p53 activation enforces lineage-specific apoptosis, producing the hypoproliferative phenotype; 4. chronic p53-driven selective pressure favors subclones with *TP53* pathway alterations or compensatory ribosome-rescuing mutations (e.g., *EIF6* in SDS); 5. once p53 is inactivated, residual translational abnormalities—altered IRES-dependent translation, rRNA modification changes, impaired proteostasis, and, in cancers with oncogenic *MYC* activation, hyperactive ribosome biogenesis—drive malignant transformation. This cascade provides the foundation for future disease-specific discussions with the cross-disease comparison summarized in **Table 1**.

**Table 1:** Cross-disease comparison of ribosomal mechanisms, driver events, and therapeutic vulnerabilities in inherited ribosomopathies and pediatric-relevant cancers.

Type	Entity	Primary ribosomal defect	Biogenesis/translation step affected	p53/MDM2 axis status	MYC/translational axis	Key therapeutic vulnerability and clinical-stage agents
Inherited	DBA	Germline heterozygous RP loss (RPS19 most common; RPL5, RPL11, RPS17)	Early pre-40S/60S assembly	Constitutive p53 activation; escape via TP53 mutations or MDM2/MDM4 alterations in clonal evolution	Variable translational reprogramming; no primary MYC axis	MDM2 antagonists to sustain p53 in non-transformed cells; Pol I inhibitors in TP53-intact secondary cancers; colorectal/osteosarcoma surveillance
	SDS	Biallelic SBDS loss; also EFL1, DNAJC21, SRP54	Late 60S maturation (SBDS/EFL1-mediated EIF6 release)	p53 activation; bifurcation into EIF6-inactivating (benign) vs. TP53-altered (pre-malignant) clones	Not a primary driver	Molecular surveillance of clonal landscape (TP53/EIF6 sequencing); allo-HSCT timing guided by clonal markers
	DC/TBDs	DKC1 loss (X-linked); also TERC, TERT, RTEL1, NHP2, NOP10	H/ACA snoRNP function → rRNA pseudouridylation (plus telomere maintenance defect)	Combined telomere + ribosome stress converges on p53	Not a primary driver	Telomere-stabilizing strategies; hematologic surveillance; anogenital/head-and-neck SCC screening
Acquired	del(5q) MDS	Somatic RPS14 haploinsufficiency	Pre-40S assembly	p53 activation in erythroid lineage drives the phenotype	Not a primary driver	Lenalidomide (selective elimination of del(5q) clone; modulates ribosome-stress landscape)
	T-ALL with RPL10 R98S	Somatic RPL10 hotspot mutation	Late 60S maturation (Nmd3-P-site trapping)	Context-dependent (often intact TP53)	↑ IRES-dependent BCL-2 translation; ↑ JAK-STAT signaling; proteotoxic stress	Venetoclax (BCL-2); JAK inhibitors or pimozide (JAK-STAT); bortezomib (proteasome); reinforced by AALL1231 trial
	T-ALL/lymphoma with RPL22 loss	Somatic RPL22 deletion (haploinsufficient tumor suppressor)	Extra-ribosomal RNA processing functions	Independent of p53 axis	↑ NF-κB signaling; ↑ Lin28B stemness axis	No direct clinical-stage agent at this node; NF-κB and Lin28B represent investigational targets
	Group 3 medulloblastoma (MYC-amplified)	MYC amplification drives hyperactive ribosome biogenesis	Pol I transcription; rRNA processing; cap-dependent translation	Frequently TP53-intact (amenable to p53 reactivation)	MYC → coordinate activation of all three RNA polymerases and eIF4F	Pol I inhibitors (CX-5461/pidnarulex); BET inhibitors (JQ1); PRMT5 inhibitors (EPZ015666, GSK3326595); mTOR (BEZ235); minnelide
	Neuroblastoma (high-risk/MYCN-amplified)	Overexpression of nucleolin (surface and nucleolar); fibrillarin dysregulation	rRNA methylation; nucleolar organization; ribosome biogenesis	Variable TP53 status; MYCN amplification predominates	MYCN-driven ribosome biogenesis dependence	Nucleolin-targeted nanotherapy (F3 peptide-doxorubicin liposomes); AS1411 aptamer; salinomycin; Pol I inhibitors in preclinical stage
	Wilms tumor (anaplastic)	Direct alterations of the TP53 surveillance axis	Downstream — checkpoint disabled rather than biogenesis primarily affected	TP53 mutations disable the nucleolar stress checkpoint	Variable	Mutant-p53 reactivators (PRIMA-1/PRIMA-1Met/APR-246); RITA (p53-MDM2 disruption); pifithrin-μ (Hsp70 inhibition)

AML, acute myeloid leukemia; DBA, Diamond–Blackfan anemia; DC/TBDs, dyskeratosis congenita/telomere biology disorders; HSCT, hematopoietic stem cell transplantation; IRES, internal ribosome entry site; MDS, myelodysplastic syndrome; PDX, patient-derived xenograft; Pol I, RNA polymerase I; RP, ribosomal protein; SCC, squamous cell carcinoma; SDS, Shwachman–Diamond syndrome; T-ALL, T-cell acute lymphoblastic leukemia.

### 3.3 Ribosome specialization and translational reprogramming

Beyond simply producing more ribosomes, cancer cells can exploit qualitative changes in ribosome composition and function. The concept of specialized ribosomes—ribosome populations with distinct protein, rRNA modification, or associated factor compositions that preferentially translate specific mRNA subsets—has gained substantial experimental support over the past decade, although its extent and functional relevance in human cancers remain areas of active investigation (11, 12). Differential incorporation of paralogue ribosomal proteins (e.g., RPL10A versus RPL10 isoforms), ribosome-associated proteins, and post-translational modifications can create functionally distinct ribosome populations tuned to specific translational programs (13, 14).

In cancer, ribosome heterogeneity may support oncogenic translation programs through several mechanisms. First, preclinical studies suggest that ribosomes with altered composition may show increased affinity for structured 5′UTRs or internal ribosome entry sites (IRES), thereby preferentially translating survival factors such as BCL-2, XIAP, and growth factor receptors under stress conditions (15, 16). Second, differential rRNA modification by changes in snoRNA-guided 2′-O-methylation or pseudouridylation alter ribosome function and may selectively affect translation of subsets of oncogenic mRNAs (12, 17–19). Third, altered translational fidelity, including read-through of stop codons and +1 frameshifting, can generate neoantigens or dysregulate proteostasis in ways that confer selective advantages (20–23).

### 3.4 Translational fidelity, stress tolerance, and metabolic coupling

Ribosomal protein mutations can alter translational fidelity, elongation dynamics, and the efficiency of translating structured mRNAs or those relying on alternative initiation mechanisms (24). In T-ALL, the recurrent *RPL10* R98S mutation has been linked to impaired late steps of 60S maturation, increased sensitivity to oxidative stress, mitochondrial dysfunction, altered IRES-dependent translation (particularly of *BCL-2* family members), and enhanced JAK-STAT signaling (15, 25, 26). Ribosome dysfunction also intersects with metabolic rewiring in cancer (27). Impaired ribosome assembly increases the burden on the proteasome and other proteostasis machinery, creating synthetic lethal opportunities with proteasome inhibition. Conversely, increased ribosome biogenesis in MYC-driven tumors creates dependency on nucleotide biosynthesis, amino acid supply, and energy metabolism, linking ribosome production to broader metabolic vulnerabilities (2, 3, 28). At the level of translation, certain cancer cells become disproportionately dependent on specific translation initiation factors such as eIF4E, that can provide additional opportunities for therapeutic targeting (29, 30).

### 3.5 Dysregulation of ribosome biogenesis and rRNA modification in cancer

Ribosome function depends on extensive co- and post-transcriptional rRNA processing and modification, including 2′-O-methylation (guided by box C/D snoRNAs) and pseudouridylation (guided by box H/ACA snoRNPs, including those containing dyskerin). Each complex consists of a guide snoRNA (60–120 nucleotides) and four core proteins: the methyltransferase fibrillarin (FBL), structural proteins NOP56 and NOP58, and the RNA-binding protein NHP2L1 (15.5K/SNU13) (31, 32). The snoRNA component base-pairs with target rRNA, positioning FBL to catalyze 2’-O-methylation at the fifth nucleotide upstream of the conserved D or D’ box motif (33). While constitutive methylation sites in the ribosome core are essential for assembly and function, facultative sites in the ribosome periphery show variability across physiological states, suggesting regulatory potential (34, 35). Both protein and RNA components of this machinery are systematically dysregulated in pediatric hematological malignancies and solid tumors (36, 37). Dysregulation of these processes has been associated with altered translational fidelity and transcript selectivity, potentially shaping oncogenic proteomes even in the absence of canonical RP mutations (12, 38).

Recent research has gone beyond just looking at overall patterns to show that specific rRNA 2′-O-methylation plays an important role in AML biology. Three lines of evidence are particularly important. First, AML1-ETO—the fusion oncoprotein generated by t(8;21)—enhances C/D box snoRNP biogenesis through a pathway involving the co-repressor AES and the DEAD-box helicase DDX21, directly linking a canonical AML driver to reprogramming of the snoRNP machinery and to altered ribosome modification profiles (39). Second, SNORD42A guides 2′-O-methylation of 18S rRNA at position U116; depletion of SNORD42A reduces this specific modification and impairs AML cell proliferation and clonogenic capacity, providing evidence that a defined methylation event—rather than a global expression change—is required for leukemic growth (40). Third, quantitative ribomethylome profiling in primary AML samples has revealed that rRNA methylation patterns are dynamic across leukemia stem-cell (LSC) and non-LSC compartments, and that specific methylation signatures associate with LSC activity and clinical outcomes, establishing rRNA modification as a regulated, rather than static, feature of leukemic cell identity (41). Together, these studies demonstrate that site-specific rRNA 2′-O-methylation can function as an oncogenic effector in AML and support the concept that the stable/core versus plastic/peripheral methylation site distinction is biologically meaningful in malignancy: constitutive core-site methylation sustains ribosome integrity, whereas variability at peripheral facultative sites can be co-opted to reprogram translational output in cancer cells (34, 35).

An important methodological caveat applies throughout this section. Reliable inference about altered rRNA methylation requires direct quantitative approaches: RiboMethSeq or Nm-seq for transcriptome-wide 2′-O-methylation profiling, SILAC-coupled mass spectrometry for ribosome composition analysis, or targeted reverse-transcriptase-at-low-dNTP (RTL)-based assays for individual sites. Expression-level data for FBL, NOP56, NOP58, or snoRNA host genes—including results from our own studies—constitute correlative evidence only and do not demonstrate that specific methylation events are altered, nor that such alterations contribute to the observed phenotypes (42, 43). Where the present review draws on expression data alone, claims are explicitly framed as hypothesis-generating rather than established observations.

Fibrillarin is an S-adenosyl-L-methionine-dependent methyltransferase comprising an N-terminal glycine-arginine-rich (GAR) domain and a C-terminal methyltransferase domain (44). Beyond rRNA modification, FBL methylates yeast histone H2A at glutamine-104 (H2AQ104, the equivalent of human Q105), regulating chromatin structure at ribosomal DNA loci (45). In acute myeloid leukemia, FBL emerges as a strong candidate mediator of leukemogenesis. A genome-wide CRISPR screen identified FBL as essential for AML survival through formation of biomolecular condensates that concentrate nucleolar components and drive hyperactive ribosome biogenesis (46). FBL is highly expressed in AML patient blasts and correlates with poor prognosis, while FBL depletion induces cell cycle arrest, differentiation, and apoptosis in leukemic cells with minimal impact on normal hematopoietic stem cells (19).

 (47) When ribosome production is disturbed—including by FBL depletion—p53 is activated through the 5S RNP–MDM2–p53 axis, mediating growth inhibition (direct FBL-depletion evidence in AML is inferred from broader pathway data rather than demonstrated in a single study) (46–48).

In pediatric B-cell precursor acute lymphoblastic leukemia (BCP-ALL), FBL overexpression is associated with a prognostic signature. Teittinen and colleagues demonstrated that elevated FBL, combined with c-MYC, SNORD35B, and SNORD46, significantly associates with disease relapse and reduced leukemia-free survival (36). FBL also plays a role in DNA damage response and chemoresistance, and FBL depletion sensitizes cancer cells to DNA crosslinking agents and causes homologous recombination defects (49). In neuroblastoma, FBL expression may influence treatment response (50, 51).

NOP56 and NOP58 are highly homologous proteins (65% sequence identity) functioning as structural scaffolds within box C/D snoRNPs (52, 53). NOP56 binds the internal C’/D’ motif while NOP58 binds the terminal C/D motif (54). NOP56 is the final core protein to enter the snoRNP complex, decisive for complex activation (55). Expression levels are subject to negative feedback regulation: depletion of NOP58 and FBL increases NOP56 production, and vice versa (56). In pediatric BCP-ALL, c-MYC-driven upregulation of NOP56 and FBL represents hyperactive ribosome biogenesis, and patients with high NOP56 at diagnosis are more likely to relapse (36). In colorectal cancer, NOP58 overexpression in 5-fluorouracil-resistant cells correlates with increased glycolysis, and NOP58 knockdown re-sensitizes cells to chemotherapy (57). SNORD35B and SNORD46 are core components of the unfavorable prognostic signature in pediatric BCP-ALL (36).

SNORD50A and SNORD50B function as tumor suppressors, encoded within SNHG5 on chromosome 6q (58). Deletion of 6q—including SNHG5—is recurrent in pediatric T-ALL (10% of cases) (59). The SNORD50A-SNORD50B snoRNA locus was deleted in 10-40% of 12 common cancers, where its loss was associated with reduced survival (60). Loss of SNORD50A and SNORD50B increased the amount of GTP-bound, active K-Ras and hyperactivated Ras-ERK1/ERK2 signaling. Given RAS pathway alterations in pediatric neuroblastoma (*ALK* mutations), juvenile myelomonocytic leukemia (*NRAS*/*KRAS*), and rhabdomyosarcoma, the SNORD50A/B-K-Ras axis may have therapeutic implications.

The proto-oncogene *c-MYC* coordinately controls all three RNA polymerases involved in ribosome production and enhances expression of ribosomal protein genes (2, 61). The MYC-driven cancers (Burkitt lymphoma, MYCN-amplified neuroblastoma, and group III medulloblastoma) require hyperactive ribosome production and are consequently vulnerable to ribosome biogenesis inhibitors (62). Complex feedback loops regulate MYC-RP interactions; while c-MYC drives ribosome production, ribosomal proteins RPL5 and RPL11 suppress c-MYC activity, but when ribosome assembly is perturbed, free RPL5/RPL11 (5S RNP) inhibit MDM2, stabilizing p53, which represses c-MYC (63). In consequence, TP53 mutations in cancer can result in unchecked c-MYC-driven ribosome biogenesis. Therapeutically, the c-MYC-ribosome axis presents multiple intervention points. Direct MYC inhibition remains challenging, though MYC-MAX heterodimerization inhibitors and MYC stability modulators are in development (64). Downstream targeting via RNA Pol I inhibition (CX-5461) or FBL inhibition provides alternatives (62, 65). In pediatric patients with MYC/MYCN-driven cancers and intact TP53, combining ribosome biogenesis inhibitors with MDM2 antagonists could synergistically activate p53 while blocking ribosome production (66, 67).

Nucleolin is among the most abundant non-ribosomal nucleolar proteins, orchestrating rDNA chromatin remodeling, RNA Pol I transcription, pre-rRNA processing, and ribosome assembly (68–70). NCL contains an N-terminal acidic domain, central RNA-binding domains, and C-terminal arginine-glycine-rich domain, enabling diverse nucleic acid and protein interactions (71). NCL localizes predominantly in the nucleolus (>90%) but also in the nucleoplasm and cytoplasm; in cancer cells and angiogenic endothelial cells, it is additionally expressed on the cell surface, a localization that is absent or rare in quiescent normal cells (72). Expression profiling of 270 AML samples demonstrated NCL overexpression in blasts compared to normal marrow (73). High *NCL* mRNA correlates with poor overall survival, and multivariate analysis indicates NCL expression is an independent marker of reduced survival. NCL overexpression sustains high protein synthesis rates required by leukemic cells. Cytoplasmic NCL regulates post-transcriptional gene expression. In chronic lymphocytic leukemia, cytoplasmic NCL stabilizes *BCL-2* mRNA, increasing BCL-2 protein and promoting cell survival (74). NCL also binds p53 mRNA 5’-UTR, preventing translation and suppressing p53 independent of MDM2 (75). Surface-localized NCL represents a tumor-selective therapeutic target, particularly in neuroblastoma. Neuroblastoma cell lines, primary tumors, and bone marrow-infiltrating cells express surface NCL (76). NCL-targeted nanocarriers, specifically F3 peptide-decorated pegylated liposomal doxorubicin, demonstrate selective cytotoxicity *in vitro* and anti-tumor efficacy in metastatic and orthotopic xenografts. The F3 peptide selectively binds surface NCL, mediating internalization of conjugated therapeutics (77). Additional NCL-targeting strategies include the AS1411 aptamer, a G-quadruplex oligonucleotide evaluated in phase II trials for renal cell carcinoma and AML, demonstrating clinical activity and acceptable safety (78, 79). Cell surface NCL binds Fas, inhibits ligand binding, and thus prevents induction of Fas-mediated apoptosis in B-cell lymphomas (80).

Dysregulation of the rRNA methylation machinery may contribute to oncogenic programs in pediatric malignancies and represents a potentially targetable vulnerability. c-MYC functions as master coordinator, upregulating FBL, NOP56, nucleolin, and snoRNA host genes, creating a mechanism sustaining hyperactive ribosome biogenesis (36, 61, 81). The tight component correlation in BCP-ALL reflects coordinate regulation, explaining why multi-component signatures outperform single biomarkers (36, 42). Altered rRNA methylation from dysregulated snoRNP activity may generate ribosomes with distinct translational properties, although direct evidence from primary pediatric tumors remains limited to correlative expression data; quantitative methylation profiling is required to establish causality (34, 35). Based primarily on preclinical and translational studies, high FBL expression and snoRNA dysregulation raise the hypothesis that altered facultative methylation sites may favor translation of selected oncogenic transcripts, potentially enhancing IRES-dependent translation of oncogenic mRNAs (BCL-2, MYC); direct evidence for this mechanism in primary pediatric tumors is, however, currently lacking (82, 83). The disruption of ribosome biogenesis triggers p53 activation in *TP53*-wild-type cells (47, 48). *TP53* mutations in relapsed ALL (10%), secondary AML from ribosomopathies, and high-risk solid tumors disable this surveillance, explaining why ribosome biogenesis dysregulation predicts poor outcomes (84, 85). A recent study examining ribosome biogenesis regulators in pediatric pre-B ALL and neuroblastoma showed that both diseases displayed impairment of nucleolar surveillance and suppression rather than upregulation of key rRNA processing effectors, suggesting distinct oncogenic programs engaging the nucleolar machinery and qualitative translational reprogramming (43). Therapeutic strategies target multiple levels: RNA Pol I inhibition blocks upstream rDNA transcription, FBL inhibition impairs methylation, and NCL-directed strategies (F3-peptide, AS1411) enable selective drug delivery, snoRNA modulation via antisense oligonucleotides, c-MYC pathway disruption, and combination approaches pairing ribosome biogenesis inhibitors with MDM2 antagonists (62, 64–67, 76, 86). Developmentally, children in active growth require high baseline ribosome biogenesis, potentially narrowing therapeutic windows. However, rapid pediatric cancer proliferation creates differential sensitivity where modest ribosome biogenesis inhibition is tolerated by slowly dividing normal tissues but is catastrophic for leukemic blasts (87).

## 4 Constitutional ribosome dysfunction and cancer predisposition

### 4.1 Diamond–Blackfan anemia: ribosomal protein haploinsufficiency

DBA is the archetypal ribosomopathy, most often caused by heterozygous loss-of-function variants in ribosomal protein genes (most commonly RPS19, followed by RPL5, RPL11, RPS17, RPL35A, and others) (88, 89). It presents in the first year of life with congenital hypoplastic anemia and variable congenital anomalies, including craniofacial, limb, and cardiac defects. Approximately 70% of DBA patients carry identifiable RP gene mutations by comprehensive genetic testing, a subset have structural rearrangements or promoter variants (90). Genotype-phenotype correlations in DBA are increasingly recognized. RPS19 mutations, the most common genotype (approximately 25% of cases), typically present as hypoplastic anemia with variable and generally milder congenital anomalies. RPL5 and RPL11 mutations are disproportionately associated with multiple congenital anomalies—including cleft palate, thumb abnormalities, and cardiac defects—and appear to confer a greater cumulative anomaly burden, although definitive genotype-specific cancer risk stratification remains preliminary (89, 90).

Large registry-based analyses demonstrate substantially elevated cancer risk in DBA (88). Data from the Diamond Blackfan Anemia Registry show markedly increased observed-to-expected ratios for all cancers combined, with particularly high relative risks for MDS, AML, colorectal carcinoma, and osteogenic sarcoma; cumulative incidence of malignancy rises significantly by mid-adulthood (88).

The model for DBA-associated cancer predisposition is best understood as a two-phase process with clonal evolution at its core (**Figure 1**) explained mechanistically in section 3.2 (27, 91, 92). Persistent translational abnormalities secondary to constitutive RP haploinsufficiency may also alter proteostasis and genomic stability in ways that contribute to transformation independently of p53 pathway escape (93, 94). The 2024 international consensus statement provides risk-adapted surveillance recommendations, including annual hematologic monitoring, colonoscopic screening from age 30 for colorectal cancer, and genotype-aware counselling (89).

### 4.2 Shwachman–Diamond syndrome: ribosome assembly defects, clonal evolution, and TP53 selection

SDS is an inherited marrow failure syndrome classically caused by biallelic SBDS mutations, with less common cases attributed to mutations in DNAJC21, EFL1, or SRP54 (95–97). Ribosome assembly defects in classical SDS are tightly linked to late steps in 60S maturation: SBDS cooperates with EFL1 to displace EIF6 from the 60S pre-ribosomal particle, enabling the final step of large-subunit maturation and export (98, 99). Loss of SBDS function impairs this step, creating a ribosome assembly checkpoint that constitutes a fitness constraint in hematopoietic stem and progenitor cells (HSPCs) (99). Compensatory clones bearing inactivating EIF6 changes partially rescue ribosome maturation and are associated with lower leukemic potential (99). In contrast, premalignant clones with TP53 pathway alterations—including *TP53* point mutations or del 17p—confer elevated leukemic potential and define a high-risk subgroup (99). Epidemiologic analyses have recently expanded the recognized neoplasia spectrum in SDS beyond MDS/AML to include lymphoid malignancies, highlighting that ribosome assembly defects may confer broader lymphoid cancer risk than previously appreciated (100).

SDS provides the clearest genetic evidence that the ribosomopathy paradox is resolved through clonal selection on the p53 axis. The bifurcation into compensatory (EIF6-inactivating, lower leukemic risk) and pre-malignant (TP53 pathway, high leukemic risk) clones is clinically actionable, and emerging as a tool for transplant timing decisions, identifying patients whose clonal landscape shifts toward the high-risk pathway before overt MDS/AML develops.

### 4.3 Dyskeratosis congenita/telomere biology disorders with ribosome-relevant components

While telomere biology disorders (TBDs) are defined by telomere maintenance defects, the causal gene *DKC1* (encoding dyskerin, stabilizing the telomerase RNA component/TERC) participates in H/ACA snoRNP biology and rRNA pseudouridylation, functionally intersecting with ribosome integrity (101–103). Dyskerin guides the pseudouridylation of specific uridine residues in 18S and 28S rRNAs, modifications that are important for ribosome structure and translational accuracy (102). Loss of dyskerin function simultaneously impairs telomere maintenance and ribosome modification, creating a compound cellular stress environment.

Clinical reviews and cohort studies consistently note elevated risks of bone marrow failure, MDS/AML, and selected solid tumors—most prominently squamous cell carcinomas of the head/neck and anogenital region—in TBDs (104–108). The connection between TBDs and ribosomopathies is one of chronic stress surveillance operating through p53 and related pathways, creating long-term evolutionary pressure in hematopoietic tissues, ultimately selecting for malignant clones with checkpoint escape (109–111).

## 5 Somatic ribosomal protein mutations and leukemogenesis

### 5.1 T-ALL

Somatic ribosomal protein mutations in T-ALL represent examples of acquired ribosome dysfunction in leukemia. Large-scale sequencing studies have identified recurrent mutations in *RPL10*, *RPL5*, *RPL22*, and *RPL11*, with aggregate frequencies of RP lesions approaching 10–20% of T-ALL cases in comprehensive pediatric series (59). These mutations are non-overlapping and mutually exclusive with one another, suggesting convergent selection on ribosome function rather than independent passenger accumulation.

The *RPL10* R98S hotspot mutation is the most extensively characterized. Functional work demonstrates that *RPL10* R98S impairs late steps of 60S maturation and creates a translational state associated with oxidative stress, mitochondrial dysfunction, increased IRES-dependent *BCL-2* translation, and enhanced JAK-STAT signaling (15). Critically, each of these proteins represents a potential therapeutic target: venetoclax can exploit *BCL-2* upregulation, JAK inhibitors or pimozide suppress the enhanced JAK-STAT signaling and proteasome inhibitors (bortezomib) exploit the increased proteotoxic stress created by ribosome dysfunction. The addition of bortezomib to backbone therapy in pediatric T-lymphoblastic leukemia and lymphoma (AALL1231) has shown clinical benefit, providing support for the relevance of proteostasis targeting in this disease (112). Interestingly, the occurrence of *RPL10* mutations in an adult malignancy—multiple myeloma MM - at sites distinct from the T-ALL R98S hotspot suggests diagnosis-specific selective pressures on translation machinery in plasma cells, which are characterized by extreme dependence on secretory protein synthesis and endoplasmic reticulum homeostasis. The induction of proteotoxic stress by targeting protein degradation with proteasome inhibitors has revolutionized the treatment of MM, and the ribosome pathway is the top downregulated KEGG pathway in bortezomib responders versus non-responders (113, 114). *RPL22*, encoding a ribosomal protein with extra-ribosomal functions in RNA processing, behaves as a haploinsufficient tumor suppressor in T-ALL models (115). Loss of one *RPL22* allele accelerates lymphomagenesis, the formation of lymphomas, through NF-κB-dependent induction of the stemness factor Lin28B, which in turn elevates *MYC* and *RAS* expression via suppression of let-7 miRNAs (115). This extra-ribosomal tumor suppressor function of *RPL22* illustrates that RP cancer biology extends beyond effects on translation.

### 5.2 Acquired ribosomopathy—del(5q) and RPS14 haploinsufficiency

The myelodysplastic syndrome with isolated del(5q) represents an acquired ribosomopathy. Functional genomics studies demonstrated that haploinsufficiency of *RPS14*, located within the commonly deleted region at 5q33.1, is the critical driver of the characteristic erythroid differentiation defect and macrocytic anemia (116). RNA interference-mediated depletion of *RPS14* in normal hematopoietic progenitors phenocopies the del(5q) erythroid failure, while forced *RPS14* expression rescues the phenotype. Gene expression signatures in del(5q) erythroid progenitors are consistent with aberrant ribosome biogenesis stress and p53 activation.

This reinforces that ribosome dysfunction can be both a primary lesion and a shaping force in clonal hematopoiesis and myeloid neoplasia and that the mechanisms identified in inherited ribosomopathies (ribosome stress/p53 activation/lineage-specific apoptosis) are recapitulated in cancer by acquired ribosomal gene haploinsufficiency. The therapeutic sensitivity of del(5q) MDS to lenalidomide—mediated through selective elimination of the del(5q) clone and restoration of normal erythropoiesis—may in part reflect manipulation of this ribosome stress landscape (117).

## 6 Ribosomal dysregulation in solid tumors

While somatic ribosomal protein mutations are best characterized in hematologic malignancies, growing evidence implicates ribosome-related pathways in the biology and potential treatment of pediatric solid tumors. In these entities, direct mutations in RP genes are less frequent than in T-ALL; instead, the oncogenic relevance of ribosome biology manifests through dysregulation of ribosome biogenesis regulators (nucleolin, fibrillarin), translational control via the MYC–ribosome axis, and the interplay between ribosomal stress signaling and the TP53 tumor suppressor pathway. The following subsections summarize current evidence for neuroblastoma, medulloblastoma, and Wilms tumor—three pediatric solid tumors in which ribosome-related mechanisms have therapeutic implications.

### 6.1 Neuroblastoma

As discussed above, NCL orchestrates multiple steps of ribosome biogenesis and is aberrantly expressed on the cell surface of neuroblastoma cells, primary tumors, and bone marrow-infiltrating neuroblasts (76). The dual expression of surface NCL on both tumor cells and angiogenic tumor endothelium makes it an attractive target for simultaneous anti-tumor and anti-vascular therapy (72, 76). Initial characterization of the F3 homing peptide demonstrated selective recognition and binding of NCL-expressing tumor cells and endothelial cells, establishing the principle of NCL-directed drug delivery (77). Subsequent studies in adult malignancies, including glioblastoma, confirmed that F3 peptide-targeted, doxorubicin-entrapping, pH-sensitive liposomal formulations achieve selective intracellular delivery and enhanced cytotoxicity in NCL-positive tumors (118, 119). Translation of this platform to neuroblastoma demonstrated that F3-targeted nanoliposomes deliver doxorubicin to NCL-positive neuroblastoma cells with cytotoxic efficacy both *in vitro* and in metastatic and orthotopic xenograft models, supporting clinical development of NCL-directed nanotherapy in this disease (76).

An independent NCL-targeting strategy exploits salinomycin, an ionophore anticoccidial antibiotic with anti-cancer stem cell activity. Originally characterized as an inhibitor of breast cancer growth through NCL-dependent mechanisms, salinomycin was subsequently shown to bind surface NCL on neuroblastoma stem cells, disrupting the NCL–CD34 promoter complex and suppressing CD34 expression—a marker of the cancer stem cell compartment (119, 120). This mechanism is of particular interest because it targets the self-renewal capacity of neuroblastoma-initiating cells rather than the bulk tumor population.

Beyond its direct roles in ribosome biogenesis and cell-surface signaling, NCL participates in non-coding RNA-mediated oncogenic circuits in neuroblastoma. The long non-coding RNA LINC01296 directly binds NCL to form a ribonucleoprotein complex that activates transcription of SRY-box transcription factor 11 (SOX11), a neural developmental regulator implicated in tumor progression (121, 122). LINC01296 is upregulated in advanced-stage compared with early-stage neuroblastoma and associates with poor patient outcomes (121). Knockdown of LINC01296 inhibits cell proliferation and migration while promoting apoptosis, suggesting that the LINC01296–NCL–SOX11 axis functions both as a prognostic biomarker and a potential therapeutic target in high-risk neuroblastoma (122).

NCL-targeting aptamers provide a further avenue for selective drug delivery. AS1411, a guanosine-rich oligonucleotide that folds into a G-quadruplex structure and binds surface NCL with high affinity, has been evaluated in phase II clinical trials in renal cell carcinoma and AML, demonstrating acceptable safety and preliminary clinical activity (78, 79). Because AS1411 localizes preferentially to NCL-positive cells, it serves as both a potential delivery vehicle for conjugated therapeutics and a diagnostic tool for identifying tumors with high surface NCL expression (123, 124). Integration of AS1411-based strategies with existing NCL-targeted nanotherapies may further improve the precision of ribosome-associated therapeutic approaches in neuroblastoma.

### 6.2 Medulloblastoma

MYC amplification defines Group 3 medulloblastoma, the subgroup with the worst prognosis, and directly drives hyperactive ribosome biogenesis through coordinate upregulation of all three RNA polymerases, ribosomal protein gene transcription, and rRNA processing factors (see section 3.5). This apparent dependence on elevated translational output may create a therapeutic vulnerability: MYC-amplified medulloblastoma cells cannot sustain proliferation when ribosome production is impaired. Importantly, MYC increases protein synthesis not only through transcriptional regulation of ribosomal gene expression but also through mTOR-dependent translational control, establishing convergent oncogenic inputs on the ribosome (125).

Several therapeutic strategies targeting the MYC–ribosome axis have shown preclinical activity in medulloblastoma. BET bromodomain inhibitors, exemplified by JQ1 (a thieno-triazolo-1, 4-diazepine compound that competitively binds to the acetyl-lysine recognition pocket of BET bromodomains), suppress MYC transcription by displacing BRD4 from super-enhancers driving MYC expression, thereby reducing downstream ribosome biogenesis and protein synthesis (126, 127). In MYC-amplified medulloblastoma models, JQ1 treatment inhibits tumor growth, providing proof-of-concept that epigenetic MYC suppression can collapse the ribosome biogenesis program (126). The dual PI3K/mTOR inhibitor BEZ235 selectively inhibits the mTOR-dependent arm of MYC-driven translation and shows activity both as monotherapy and in combination with conventional chemotherapy in medulloblastoma models (128, 129). Notably, the combination of JQ1 and mTOR inhibitors produces synergistic anti-tumor effects, consistent with simultaneous blockade of transcriptional and translational inputs converging on ribosome output (130).

Protein arginine methyltransferase 5 (PRMT5) has emerged as an additional MYC-dependent regulator in Group 3 medulloblastoma. PRMT5 expression correlates with MYC amplification and poor prognosis, and the selective PRMT5 inhibitor EPZ015666 significantly inhibits proliferation and survival of MYC-amplified medulloblastoma cells while inducing G1–S cell-cycle arrest (131). MYC-amplified cells display enhanced sensitivity to PRMT5 inhibition compared with non-amplified counterparts, reinforcing the concept that PRMT5 operates in the pathway downstream of MYC (131). The clinical-stage PRMT5 inhibitor GSK3326595, currently in phase II evaluation in MYC-positive breast cancer (NCT04676516), represents a candidate for therapeutic exploration in MYC-driven medulloblastoma (132).

More recently, the triptolide prodrug minnelide has been shown to target MYC at multiple levels, reducing both MYC transcription and MYC protein stability in medulloblastoma models (133). Minnelide inhibits tumor growth and leptomeningeal dissemination—a major clinical challenge in relapsed medulloblastoma—and augments the efficacy of adjuvant chemotherapy, improving survival in preclinical models. These preclinical findings support further evaluation of minnelide as a potential component to combination strategies targeting the MYC–ribosome biogenesis axis in high-risk medulloblastoma (133).

### 6.3 Wilms tumor

TP53 alterations, including missense mutations, deletions, and loss of heterozygosity, are among the most prognostically significant molecular events in Wilms tumor, consistently associated with anaplastic histology, chemoresistance, and poor outcomes (134). Within the framework of ribosome biology, TP53 mutations disable the nucleolar stress surveillance pathway (5S RNP–MDM2–p53 axis) that would otherwise restrict proliferation of cells with aberrant ribosome biogenesis (see section 3.2). In Wilms tumors, loss of this checkpoint may enable tolerance for the high ribosomal output required by rapidly proliferating embryonic cells, paralleling the clonal evolution dynamics described in inherited ribosomopathies.

A comprehensive bioinformatic analysis of the TP53 signaling pathway in Wilms tumor identified several candidate therapeutic agents targeting distinct nodes of TP53 biology (135). PRIMA-1 and its methylated analog PRIMA-1^Met^ (APR-246) reactivate mutant p53 by promoting proper protein folding and restoring transcriptional transactivation, inducing apoptosis in cancer cells carrying TP53 mutations (136). Pifithrin-μ, an inhibitor of the p53–heat shock protein 70 interaction, has been proposed as an adjuvant agent to enhance the efficacy of conventional chemotherapeutics (137, 138). RITA, a small molecule that disrupts the p53–MDM2 interaction and thereby stabilizes wild-type p53, demonstrates anti-tumor activity across pediatric embryonal tumors, including medulloblastoma and neuroblastoma, independently of TP53 mutational status (139).

Taken together, the evidence from neuroblastoma, medulloblastoma, and Wilms tumor illustrates that ribosome-related pathways are therapeutically relevant across pediatric solid tumors, even in the absence of direct ribosomal protein coding mutations. Conceptually, reactivation of the p53 checkpoint could restore aspects of nucleolar surveillance in tumors with ribosomal pathway dysregulation, although this remains to be tested directly in Wilms tumor models.

## 7 Targeting ribosome biogenesis and translation

**Figure 2:**
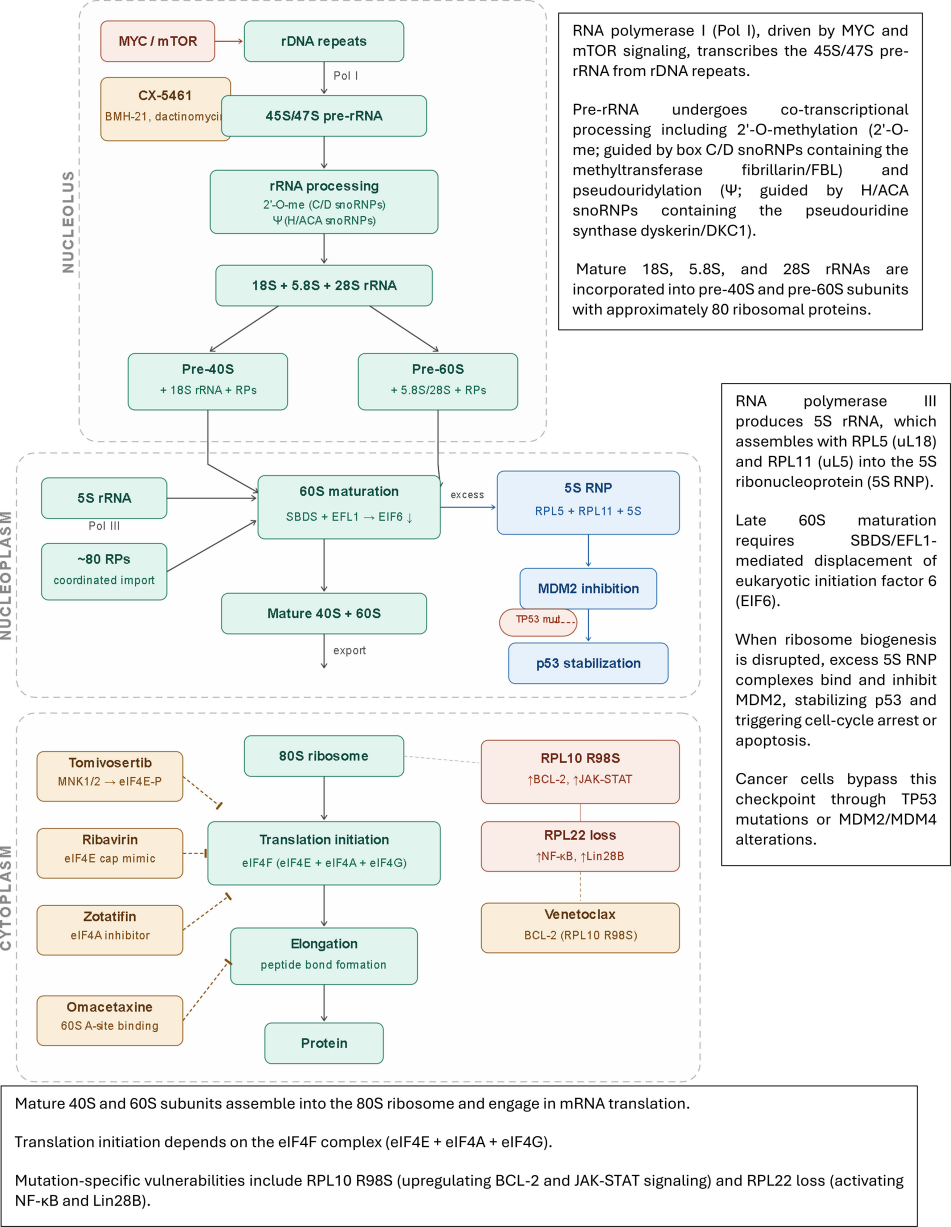
The ribosome biogenesis pathway with points for therapeutic intervention. Therapeutic targets: Pol I inhibitors (CX-5461/pidnarulex, BMH-21, dactinomycin); eIF4E cap mimic (ribavirin); MNK1/2 inhibitor (tomivosertib); eIF4A inhibitor (zotatifin); translation elongation inhibitor (omacetaxine/homoharringtonine, binding the 60S A-site); and BCL-2 antagonist (venetoclax, exploiting RPL10 R98S–driven BCL-2 dependence).

The overview of ribosomal machinery and druggable vulnerabilities are presented in **Figure 2**.

### 7.1 RNA polymerase I inhibition and nucleolar stress induction

RNA polymerase I-directed strategies aim to exploit the elevated ribosome biogenesis requirement of cancer cells and trigger nucleolar stress through 5S RNP-mediated MDM2 inhibition and p53 stabilization. CX-5461, a first-in-class selective rDNA transcription inhibitor, has been evaluated in a first-in-human phase I dose-escalation study in advanced hematologic malignancies, establishing dosing and demonstrating proof-of-principle clinical activity (62). Studies in AML models show that CX-5461 activates the DNA damage response (ATM/ATR) in addition to nucleolar stress pathways and synergizes with venetoclax and other agents targeting the apoptotic machinery (87, 140). An important observation is that therapeutic windows for Pol I inhibition depend on oncogene-driven Pol I addiction, defective checkpoints, or heightened baseline stress loads. Tumors with TP53 mutations may have attenuated p53-dependent responses to nucleolar stress induction, while paradoxically the replication stress and DNA damage components of CX-5461 action may retain activity. NPM1-mutated AML represents a particularly promising disease, because NPM1 localizes aberrantly in the cytoplasm, and low-dose actinomycin D (dactinomycin) exploits nucleolar stress sensitivity of this subtype (141).

### 7.2 Translation initiation factor targeting

Targeting translation initiation factors downstream of ribosome biogenesis has emerged as a distinct and complementary strategy. eIF4E, the mRNA cap-binding protein and rate-limiting component of the eIF4F complex, is frequently overexpressed or hyperactivated in AML and other malignancies. Ribavirin, a cap-mimetic that interferes with eIF4E-dependent mRNA export and translation, showed proof-of-principle clinical activity in eIF4E-high AML patients (29). The MNK1/2 kinases, which phosphorylate eIF4E at Ser209, represent a pharmacologically accessible node for dampening pro-survival translation without complete eIF4F complex disruption; MNK inhibitors (e.g., tomivosertib) show preclinical activity in AML, particularly in combination with venetoclax (30).

eIF4A, the DEAD-box RNA helicase component of eIF4F that unwinds structured 5′UTRs to enable scanning, is another compelling target. Rocaglate-class compounds (silvestrol; clinical-stage zotatifin) lock eIF4A onto mRNA and selectively collapse translation of 5′-TOP and other structured leader-containing mRNAs, many of which encode oncoproteins with short half-lives (142, 143). Targeting this oncogenic mRNAs represents a potentially favorable therapeutic target for aggressive leukemias driven by MYC, BCL-2, and other short-lived oncoproteins.

## 8 Clinical trials overview

Mechanistic insights linking ribosomal dysfunction to leukemogenesis and tumorigenesis have begun to translate into clinical trials. Although no ribosome-directed agent has yet received regulatory approval specifically for a pediatric cancer indication, early-phase studies in both adult and pediatric populations test agents that directly or indirectly target ribosome biogenesis, translational control, or ribosomal stress pathways (**Tables 2**, **3**). The following section summarizes these trials by mechanism of action and discusses their relevance to pediatric oncology.

**Table 2:** Clinical trials targeting ribosome biology and translation in pediatric malignancies.

NCT identifier	Disease/phase	Agent(s)	Ribosome/translation target	Status	Key findings/notes
NCT06855810	Newly diagnosed pediatric T-ALL (Phase II/III)	Homoharringtonine + venetoclax (± dasatinib)	Translation elongation inhibition: HHT binds the A-site cleft of the 60S ribosomal subunit, blocking peptide bond formation	Recruiting	Combines ribosome-targeted translation inhibition with BCL-2 antagonism, rationale includes HHT-mediated Mcl-1 depletion synergizing with venetoclax (144–146).
NCT06742463	R/R T-ALL/LBL, age ≥14 y (Phase II)	Venetoclax + homoharringtonine (+ cytarabine, G-CSF)	Translation elongation inhibition (60S A-site); concurrent Mcl-1 reduction via translational suppression	Recruiting	Pilot data in adult R/R ETP-ALL: CRc 100% (7/7), MRD negativity 100% after 2 cycles; 85.7% bridged to allo-HSCT (147).
NCT03165851	Pediatric AML (retrospective/observational)	Homoharringtonine	Translation elongation inhibition (60S A-site)	Completed	Retrospective evaluation of HHT-containing regimens in pediatric AML, limited published data (148).

AML, acute myeloid leukemia; CRc, composite complete remission; ETP-ALL, early T-cell precursor ALL; HHT, homoharringtonine; HSCT, hematopoietic stem cell transplantation; LBL, lymphoblastic lymphoma; R/R, relapsed/refractory; T-ALL, T-cell acute lymphoblastic leukemia; MRD, minimal residual disease.

**Table 3:** Clinical trials targeting ribosome biogenesis and translational control in adult malignancies with pediatric translation perspectives.

NCT identifier	Disease/Phase	Agent(s)	Ribosome/translation target	Status	Key findings	Pediatric translation perspectives
NCT02719977	Advanced solid tumors (Phase I)	Pidnarulex (CX-5461)	RNA Pol I inhibition; G-quadruplex stabilization; nucleolar stress induction.	Completed	First-in-human Pol I inhibitor trial, dose-escalation established recommended phase II dose, clinical activity in BRCA/HRD-deficient tumors (149).	HRD mutations rare in childhood cancers, however, MYC-driven tumors (neuroblastoma, medulloblastoma) may respond via nucleolar stress mechanisms.
NCT04890613	HRD solid tumors (BRCA/PALB2) (Phase I)	Pidnarulex (CX-5461)	rDNA transcription/replication stress linked to ribosome biogenesis.	Recruiting	No published results.	HRD-driven tumors uncommon in children, pediatric relevance limited to rare BRCA-associated malignancies.
NCT05425862	Metastatic CRPC (Phase I)	Pidnarulex + talazoparib	Ribosome biogenesis/rDNA stress + PARP inhibition (DDR combination).	Suspended	Enrollment suspended to assess supplementary non-clinical data (150).	Combination of nucleolar stress + DDR inhibition represents a transferable concept.
NCT06606990	Solid tumors with metastatic disease (Phase I)	Pidnarulex (CX-5461)	rDNA transcription/replication stress.	Recruiting	No published results (151).	Broad solid tumor eligibility, pediatric solid tumors share ribosome biogenesis dependence.
NCT07069699	Aggressive B-cell NHL (Phase Ib/II)	Pidnarulex (CX-5461)	Ribosome biogenesis/nucleolar axis.	Recruiting	No published results.	Burkitt lymphoma with MYC/BCL2/BCL6 rearrangements occurs in children.
NCT07147231	Refractory MSS colorectal cancer (Phase I/II)	Pidnarulex ± anti-PD-1	rDNA/nucleolar stress ± immune combination.	Not yet recruiting	No published results.	CRC very rare in children, limited direct translation but immunotherapy combination concept is transferable.
NCT00780663	Neuroendocrine/carcinoid tumors (Phase II)	Quarfloxin (CX-3543)	rRNA biogenesis inhibition via rDNA G-quadruplex disruption.	Completed	No published results.	Neuroendocrine tumors extremely rare in children.
NCT00955786	Advanced solid tumors/lymphoma (Phase I)	Quarfloxin (CX-3543)	rRNA biogenesis inhibition via rDNA G-quadruplex/nucleolin disruption.	Completed	Preliminary dose-escalation data presented (abstract), no full publication (152).	Solid tumors and lymphoma relevant to pediatric oncology.
NCT02440568	AML (Phase I/II)	Omacetaxine mepesuccinate + 7 + 3 induction	Translation elongation inhibition (ribosomal A-site binding).	Terminated	Results published on trial registry, likely terminated due to insufficient superiority over standard induction.	AML is the second most common pediatric leukemia, HHT/omacetaxine-based regimens actively studied in pediatric T-ALL.
NCT00559091	AML M4/M5 (Phase II)	Ribavirin	eIF4E functional targeting (m^7^G cap mimic).	Completed	Proof-of-principle: clinical responses in eIF4E-high AML, biological correlates confirmed eIF4E pathway modulation (29).	M4/M5 AML present in children.
NCT02073838	R/R AML M4/M5 or high eIF4E (Phase II)	Ribavirin + vismodegib ± decitabine	eIF4E cap mimic + hedgehog/epigenetic modulation.	Completed	Published results on combination tolerability and biological activity (153, 154).	Similar rationale as NCT00559091, combination approach may enhance efficacy in pediatric eIF4E-high AML.
NCT00903708	Advanced cancers (Phase I)	LY2275796 (eIF4E ASO)	Direct eIF4E downregulation (antisense oligonucleotide).	Completed	No published results.	Broad cancer eligibility.
NCT01675128	Advanced solid tumors/irinotecan-refractory CRC (Phase I/II)	ISIS 183750 (eIF4E ASO) + irinotecan	eIF4E downregulation (antisense) + chemotherapy.	Completed	Results published, combination evaluated in CRC cohort (155).	CRC rare in children (often hereditary), eIF4E antisense may be applicable to pediatric tumors with eIF4E overexpression.
NCT04092673	Selected advanced solid tumors (Phase I/II)	Zotatifin (eFT226)	Translation initiation blockade (eIF4A RNA helicase inhibitor).	Recruiting	No published results (156).	eIF4A mechanism relevant to MYC-driven pediatric cancers pending dedicated studies.
NCT02605083	Advanced solid tumors (Phase I/II)	Tomivosertib (eFT508)	MNK1/2 kinase inhibition → eIF4E Ser209 phosphorylation blockade.	Terminated	Preliminary abstract data on tolerability, formal results not published.	MNK1/2–eIF4E axis may be relevant in selected pediatric cancers.
NCT03616834	Solid tumors on/after anti-PD-(L)1 therapy (Phase II)	Tomivosertib + PD-1/PD-L1 inhibitor	MNK1/2 inhibition + immunotherapy.	Completed	Abstract data, combination aimed at overcoming checkpoint resistance (157–159).	MNK1/2–eIF4E axis and checkpoint inhibition combination strategy.
NCT04261218	Advanced breast cancer (Phase I)	Tomivosertib + paclitaxel	MNK1/2 inhibition + cytotoxic chemotherapy.	Completed	No published results.	MNK1/2–eIF4E axis and chemotherapy combination strategy.
NCT01332786	R/R AML (Phase I)	Tigecycline (IV)	Mitochondrial translation inhibition (mitoribosome targeting).	Completed	Preclinical rationale confirmed: AML cells depend on mitochondrial translation, clinical pharmacokinetics characterized (160).	AML relevant to pediatric hematology, tigecycline is an approved antibiotic with known pediatric safety profile, facilitating drug repurposing.

AML, acute myeloid leukemia; ASO, antisense oligonucleotide; CRC, colorectal cancer; CRPC, castration-resistant prostate cancer; DDR, DNA damage response; eIF, eukaryotic initiation factor; HRD, homologous recombination deficiency; MNK, MAP kinase-interacting kinase; MSS, microsatellite-stable; NHL, non-Hodgkin lymphoma; Pol I, RNA polymerase I.

### 8.1 Translation elongation inhibitors: homoharringtonine and omacetaxine

Homoharringtonine (HHT; omacetaxine mepesuccinate in its semisynthetic form) is the ribosome-targeted agent closest to pediatric clinical application. HHT binds the A-site cleft of the 60S ribosomal subunit and blocks the initial elongation step of protein synthesis, preferentially depleting short-lived oncoproteins including Mcl-1, MYC, and cyclin D1 (144, 161). Omacetaxine is FDA-approved for tyrosine kinase inhibitor-resistant chronic myeloid leukemia and has been studied in adult AML (NCT02440568), although that trial was terminated, likely due to insufficient superiority over standard induction. In the pediatric setting, two actively recruiting trials are testing HHT-based combinations in T-ALL: NCT06855810 (HHT + venetoclax ± dasatinib in newly diagnosed pediatric T-ALL) and NCT06742463 (venetoclax + HHT + cytarabine + G-CSF in relapsed/refractory T-ALL/LBL, age ≥14 years). HHT-mediated translational suppression depletes Mcl-1, the principal resistance factor to the BCL-2 antagonist venetoclax, creating synthetic lethality at the level of the mitochondrial apoptotic machinery (161). Pilot data from the adult R/R ETP-ALL cohort demonstrated a composite complete remission rate of 100% (7/7 patients) with MRD negativity in all patients after two cycles, and 85.7% of patients successfully bridged to allogeneic HSCT (147). A retrospective evaluation of HHT-containing regimens in pediatric AML (NCT03165851) has been completed, though published data remain limited.

### 8.2 RNA polymerase I inhibition and nucleolar stress: pidnarulex (CX-5461) and quarfloxin

The RNA polymerase I inhibitor pidnarulex (CX-5461) is the most extensively studied agent targeting the rDNA transcription–ribosome biogenesis axis. Originally developed as a selective Pol I inhibitor that triggers nucleolar stress and activates the 5S RNP–MDM2–p53 checkpoint, CX-5461 was subsequently recognized to also function as a G-quadruplex stabilizer, inducing replication stress and DNA damage preferentially in homologous recombination-deficient (HRD) cells (157). The first-in-human phase I trial in advanced hematologic malignancies established dosing and demonstrated proof-of-principle clinical activity (62). Currently, six trials are evaluating pidnarulex across solid tumors (NCT02719977, NCT04890613, NCT06606990), castration-resistant prostate cancer in combination with talazoparib (NCT05425862, suspended due to additional non-clinical assessments), microsatellite-stable colorectal cancer with optional anti-PD-1 (NCT07147231), and aggressive B-cell non-Hodgkin lymphoma (NCT07069699). The aggressive B-NHL trial is of potential pediatric translational interest, as MYC-rearranged Burkitt lymphoma occurs in children and is characterized by extreme dependence on ribosome biogenesis.

Quarfloxin (CX-3543), a predecessor compound that disrupts rRNA biogenesis through rDNA G-quadruplex and nucleolin interactions, was evaluated in two completed trials: a phase II study in neuroendocrine tumors (NCT00780663) and a phase I dose-escalation in advanced solid tumors and lymphoma (NCT00955786). Neither trial has produced full peer-reviewed publications, and the quarfloxin program has been superseded by pidnarulex. Nevertheless, the quarfloxin experience contributed to the understanding of nucleolar targeting and the design of subsequent CX-5461 studies.

For pediatric oncology, the potential of Pol I inhibitors extends beyond HRD-driven tumors. MYC- and MYCN-amplified cancers (Group 3 medulloblastoma, high-risk neuroblastoma, Burkitt lymphoma) exhibit dependence on elevated Pol I–driven rRNA synthesis. Preclinical data suggest that these tumors are particularly sensitive to nucleolar stress induction, especially when TP53 signaling remains intact (62, 87). Pediatric-specific trials of Pol I inhibitors remain an unmet need.

### 8.3 eIF4E-directed strategies

Targeting the mRNA cap-binding protein eIF4E, the rate-limiting component of the eIF4F translation initiation complex, has been approached through cap mimicry (ribavirin) and antisense downregulation (LY2275796, ISIS 183750). The pediatric relevance of eIF4E centers on AML, where eIF4E overexpression may represent a target. The proof-of-principle phase II trial of ribavirin monotherapy in eIF4E-high AML M4/M5 (NCT00559091) demonstrated clinical responses and confirmed biological modulation of the eIF4E pathway (120). A subsequent combination trial adding vismodegib and decitabine to ribavirin in relapsed/refractory eIF4E-high AML (NCT02073838) has been completed with published tolerability data (162). Two antisense approaches—LY2275796 (NCT00903708) and ISIS 183750 combined with irinotecan (NCT01675128)—completed enrollment but produced limited published clinical data.

### 8.4 eIF4A inhibition: zotatifin

Zotatifin (eFT226), a clinical-stage rocaglate-class eIF4A inhibitor that collapses translation of structured 5′UTR-containing oncogenic mRNAs, is under evaluation in two recruiting trials: a phase I/II dose-escalation in selected advanced solid tumors (NCT04092673) and a phase II study in ER-positive endometrial and ovarian cancers (NCT03675893). While the current trial populations are adult-only and the enrolled tumor types do not overlap with pediatric malignancies, the eIF4A mechanism is relevant to MYC-driven pediatric cancers, although current clinical support comes from adult-only studies. Rocaglate compounds selectively suppress translation of 5′-TOP and structured-leader mRNAs encoding ribosomal proteins, MYC, and BCL-2—all critical dependencies in medulloblastoma, neuroblastoma, and aggressive lymphomas. Pediatric-dedicated evaluation of eIF4A inhibitors may be justified if adult safety, pharmacokinetic, and biomarker data will support this mechanism.

### 8.5 MNK1/2 kinase inhibition: tomivosertib

The preclinical rationale for MNK1/2 inhibition in pediatric cancers rests on the role of eIF4E phosphorylation in sustaining translation of oncogenic mRNAs under stress conditions. Tomivosertib (eFT508), a selective MNK1/2 inhibitor that blocks phosphorylation of eIF4E at Ser209 and thereby attenuates translation of a specific subset of pro-survival and immune-evasion mRNAs, has been tested in three adult trials: a dose-escalation/expansion in advanced solid tumors (NCT02605083, terminated), a combination with PD-1/PD-L1 checkpoint inhibitors in immunotherapy-refractory tumors (NCT03616834), and a combination with paclitaxel in advanced breast cancer (NCT04261218). At present, the pediatric relevance of MNK1/2 inhibition remains largely hypothesis-driven and supported indirectly by adult and preclinical data.

### 8.6 Mitochondrial translation inhibition: tigecycline

Tigecycline, a glycylcycline antibiotic that inhibits the mitochondrial ribosome (mitoribosome) and suppresses mitochondrial protein synthesis and oxidative phosphorylation, was evaluated in a phase I study in relapsed/refractory AML (NCT01332786) (160). Preclinical work demonstrated that AML cells, which depend on elevated mitochondrial biogenesis, are selectively sensitive to mitochondrial translation blockade. Because tigecycline is an approved antibiotic with a known pediatric safety profile, it may represent a candidate for repurposing in pediatric AML, although clinical feasibility and antileukemic activity in children remain unproven.

### 8.7 Pediatric translation potential

While the preceding subsections describe the landscape of ribosome-directed clinical trials by mechanism, the individual agents differ substantially in their realistic prospects for pediatric clinical translation. We propose a three-tier prioritization framework to guide future pediatric development.

Tier 1 — Actively evaluated in pediatric populations. Homoharringtonine/omacetaxine is the only ribosome-targeted agent currently in pediatric-specific clinical trials. Two actively recruiting studies (NCT06855810, NCT06742463) test HHT in combination with venetoclax in T-ALL/LBL, and one retrospective pediatric AML study (NCT03165851) has been completed. The mechanistic rationale—translational depletion of short-lived oncoproteins (Mcl-1, MYC) synergizing with BCL-2 antagonism—is well-established, and adult pilot data in ETP-ALL demonstrated high response rates.

Tier 2 — Strong rationale and mature adult data supporting dedicated pediatric evaluation. Three agents in this tier deserve priority consideration. Pidnarulex (CX-5461): the aggressive B-NHL trial (NCT07069699) includes MYC-rearranged lymphomas that overlap biologically with pediatric Burkitt lymphoma, and the mechanism is directly relevant to MYC/MYCN-amplified pediatric tumors (Group 3 medulloblastoma, high-risk neuroblastoma) with intact TP53. Tigecycline: an approved antibiotic with established pediatric safety profile, supported by adult phase I data in AML and a clear mitochondrial translation dependency in leukemic blasts. Venetoclax: already integrated into pediatric oncology, with its synergy with HHT operating at the ribosome level through Mcl-1 depletion.

Tier 3 — Mechanism-relevant but currently distant from pediatric translation. eIF4A inhibitors (zotatifin), MNK1/2 inhibitors (tomivosertib), and eIF4E antisense or cap-mimic agents are currently evaluated in adult-only tumor types—breast, endometrial, ovarian, colorectal—that do not occur or are extremely rare in children. Pediatric evaluation of these mechanisms will depend on maturation of adult safety and pharmacokinetic data and on the identification of biomarker-selected pediatric populations (for example, MYC-amplified tumors for eIF4A inhibitors, high-eIF4E AML subtypes for eIF4E-directed agents). The biological rationale for ribosome-directed therapies is often stronger in pediatric cancers than in the adult indications currently under study, and dedicated pediatric trials are needed to translate this rationale into clinical benefit.

## 9 Conclusions

Constitutional defects in ribosomal proteins and assembly factors, and somatic ribosomal gene mutations in cancers, share a common mechanistic cascade that explains both the hypoplastic phenotypes of inherited ribosomopathies and their paradoxical predisposition to cancer, and provides conceptual convergence with somatic ribosomal alterations in various entities (**Table 1**). Ribosome dysfunction acts as both a driver of oncogenesis and a therapeutic vulnerability, but clinical translation particularly in pediatric populations remains at an early stage. Several knowledge gaps limit the translation of ribosome biology insights into clinical practice.

Ribosome biogenesis inhibitors have a narrower therapeutic window in growing children, whose normal tissues require higher baseline ribosome output; differential sensitivity between rapidly proliferating tumor cells and slowly dividing normal tissues nevertheless provides a dosing window. The pediatric cancer spectrum overlaps incompletely with adult trial-eligible diagnoses—most adult ribosome-targeted studies enroll breast, prostate, endometrial, or colorectal cancers with no pediatric counterpart—requiring dedicated pediatric trial designs rather than age extensions. The often decades-long latency between ribosomopathy diagnosis and malignant transformation further complicates prospective surveillance and early-intervention trial design in inherited syndromes.

A fundamental unresolved question is why some RP mutations act as drivers in specific lineages while others appear broadly tumor suppressive across tissues. This likely reflects lineage-specific translational programs, but direct evidence from primary human cancer samples remains limited. The molecular triggers that determine whether clonal evolution in SDS or DBA follows the compensatory (EIF6-type) or pre-malignant (TP53-type) pathway remain to be defined. Whether ribosome composition, rRNA modification profiles, or nucleolar stress biomarkers can predict response to ribosome-directed therapies also remains an open question. Understanding the RP function is essential for predicting effects of specific ribosomal vulnerabilities.

Actionable research priorities include: pediatric phase I/II trials of pidnarulex (CX-5461) in MYC/MYCN-amplified, TP53-intact tumors; prospective molecular monitoring in SDS and DBA cohorts via serial TP53/EIF6 sequencing to guide transplant timing; preclinical eIF4A inhibitor evaluation in patient-derived xenograft models of MYC-driven tumors; and tigecycline repurposing in refractory pediatric AML.

Future progress will depend on defining ribosome-specific translational programs, improved molecular risk stratification in inherited syndromes, and biomarker-guided translation of ribosome-directed treatments in clinical trials.
